# Escaping from, moving towards, following a path, squeezing through: lots of opportunities for moving cells

**DOI:** 10.1186/1478-811X-8-25

**Published:** 2010-09-07

**Authors:** Paola Chiarugi

**Affiliations:** 1 Department of Biochemical Sciences, University of Florence, Tuscany Tumor Institute and "Center for Research, Transfer and High Education DenoTHE", Viale Morgagni 50, 50134 Firenze, Italy

## 

Eukaryotic cells move within the surrounding environment essentially for two reasons: the necessity to reach a predetermined site or the hostility of the primitive site. Moving in the direction of an attractive site or factor is typical for embryonic movements and metastatic dissemination of cancer cells and motility strategies are very similar for both categories. Activation of an epigenetic process called epithelial mesenchymal transition (EMT) is indeed characteristic of embryonic development, of fibrotic or regeneration processes, and of the spreading of cancer cells from their primitive origin [1,2]. The most aggressive cancers have developed a further program of cellular plasticity that is very useful to adapt to particular environmental changes, i.e. the mesenchymal amoeboid transition (MAT). This additional strategy is associated with the deregulation of important oncosuppressor pathways and the hyperexpression of oncogenes, especially those linked to the activation of the Rho GTPase family [3]. The choice of migration styles enables cells to use *ad hoc *mesenchymal or amoeboid modes of motility and grants to cells of aggressive cancers the ability to move in environments with different structural characteristics using either matrix proteases to degrade the extracellular matrix (ECM) or squeezing between its gaps. This adaptability of motility styles to the environment is currently considered to be the main reason for the failure of clinical trials testing protease inhibitors in patients with metastatic cancers. Brabek et al. [4] review in this special issue the role of matrix stiffness and composition for plasticity of cancer cell motility, while Parri and Chiarugi [5] focus on the role of Rho GTPases for the *ad hoc *switch between different motility strategies. The interest of molecular biologists is particularly focussed on this family of GTPases and their regulators as targets for an effective antimetastatic therapy. Indeed, instead of inhibiting a specific motility mechanism, it would be preferable to target the adaptation skills of cancer cells to the tumor microenvironment.

This microenvironment is indeed a mandatory element for the regulation of cell motility [6]. Three key factors are affecting the shift between modes of motility: stiffness and composition of ECM (Brabek et al., this issue [4]), intratumoral hypoxia [7] and the cellular stromal counterpart of the tumor mass (Calorini and Bianchini, this issue, [8]). The latter is composed of several cell types, with fibroblasts, macrophages and endothelial cells being the most relevant for tumor progression towards a motile/aggressive phenotype. Calorini and Bianchini [8] review the role of cancer associated fibroblasts and macrophages while Brabek et al. focus on endothelial cells [4]. Cancer-associated fibroblasts (CAFs) are engaged in a bidirectional interplay with cancer cells [9]. CAFs secrete large amount of soluble factors affecting tumor progression toward a more malignant and motile phenotype. Indeed CAFs activate a pro-inflammatory route [10], likely leading cancer cells to activate the EMT motility program [11]. On the other hand, malignant cells increase the expression of other soluble factors, thereby leading to the "activation" of stromal fibroblasts. These activated fibroblasts increase their contractility, their secretion of large amount of ECM proteins (thereby changing the ECM composition), as well as their secretion of factors affecting the EMT of cancer cells. Cancer-associated macrophages (CAMs) infiltrate the cancer mass, being attracted by tumor secreting factors. CAMs show several intermediate levels of activation in response to these factors, although they all are of the M2-subtype, that is incapable of killer and antigen presenting activities, but able to affect the malignancy and motility of cancer cells [12].

The nervous system also plays an important role in cell motility, for two reasons: the secretion of neurotransmitters which also act as motility factors and the contribution of an alternative escaping way to migrating cells, commonly called perineural invasion. In this special issue Voss and Entschladen review this aspect with a particular focus on the role of cathecolamine and stress mediators on tumoral cell motility [13].

As mentioned at the beginning, a second reason for cells to move is the escape from an hostile ambiente, for example due to the scarcity of growth factors (chemotaxis), due to the presence of improper ECM (aptotaxis and durotaxis), because of the accumulation of toxic or pro-oxidant factors (escaping from primitive tumoral or inflamatory sites) or to escape oxygen or nutrient deprivation (hypoxia and ischemia). De Donatis et al. [14] focus their review on the role of growth factor gradients as regulators of a motile phenotype in which cells aim to reach a definite growth factor concentration that is suitable for cell duplication. In this context, the motile and proliferative phenotypes are mutually exclusive and the review of De Donatis et al. clarifies the role of growth factor receptor clustering and internalization in the choice between migration and duplication. While chemotaxis and durotaxis are detailed by Brabek et al. [4], the role of a pro-oxidant and/or low oxygen environment in the regulation of cell motility has been recently reviewed by Pani and Chiarugi [15].

Far from being exhaustive, this special issue focused on cell motility aims to underscore the fertility of the current research efforts in this field, as well as highlighting key questions that still are awaiting definitive answers.
